# Enhancing rice ecological production: synergistic effects of wheat-straw decomposition and microbial agents on soil health and yield

**DOI:** 10.3389/fpls.2024.1368184

**Published:** 2024-08-08

**Authors:** Yanfang Wen, Yangming Ma, Ziniu Wu, Yonggang Yang, Xiaojuan Yuan, Kairui Chen, Yongheng Luo, Ziting He, Xinhai Huang, Pengxin Deng, Congmei Li, Zhiyuan Yang, Zongkui Chen, Jun Ma, Yongjian Sun

**Affiliations:** ^1^ Crop Ecophysiology and Cultivation Key Laboratory of Sichuan Province, Sichuan Agricultural University, Wenjiang, China; ^2^ State Key Laboratory of Crop Gene Exploration and Utilization in Southwest China, Sichuan Agricultural University, Chengdu, China

**Keywords:** microbial treatment, rice yield, microbial combination, *Bacillus subtilis*, *Trichoderma harzianum*

## Abstract

**Aims:**

This study evaluated the impact of wheat straw return and microbial agent application on rice field environments.

**Methods:**

Using Rice variety Chuankangyou 2115 and a microbial mix of *Bacillus subtilis* and *Trichoderma harzianum*. Five treatments were tested: T_1_ (no straw return), T_2_ (straw return), T_3_, T_4_, and T_5_ (straw return with varying ratios of *Bacillus subtilis* and *Trichoderma harzianum*).

**Results:**

Results indicated significant improvements in rice root length, surface area, dry weight, soil nutrients, and enzyme activity across T_2_-T_5_ compared to T_1_, enhancing yield by 3.81-26.63%. T_3_ (50:50 microbial ratio) was optimal, further increasing root dry weight, soil enzyme activity, effective panicle and spikelet numbers, and yield. Dominant bacteria in T_3_ included *MBNT15*, *Defluviicoccus*, *Ro*kubacteriales, and *Latescibacterota*. Higher *Trichoderma harzianum* proportions (75% in T_5_) increased straw decomposition but slightly inhibited root growth. Correlation analysis revealed a significant positive relationship between yield and soil microorganisms like *Gemmatimonadota* and *Firmicutes* at the heading stage. Factors like dry root weight, straw decomposition rate post-jointing stage, and elevated soil enzyme activity and nutrient content from tiller to jointing stage contributed to increased panicle and spikelet numbers, boosting yield.

**Conclusion:**

The optimal *Bacillus subtilis* and *Trichoderma harzianum* ratio for straw return was 50:50, effectively improving soil health and synergizing high rice yield with efficient straw utilization.

## 1 Introduction

Rice-wheat rotation, a typical flood and drought rotation pattern, is mainly distributed in the Yangtze River basin in China (Liu and Li, 2017). As a by-product of agricultural production, crop straw is rich in nutrients valuable for crop growth and reuse, such as organic carbon, nitrogen (N), phosphorus (P), and potassium (K), thereby aiding the improvement of soil quality (Song et al., 2018; Jin et al., 2020). However, returning straw to the field directly can result in incomplete decay, the propagation of pathogens and pests, and the subsequent reduction in crop yield (Song et al., 2023). As such, how to mitigate the adverse effects of straw returning has become a key area of research. The presence of beneficial microorganisms in soil supports the use of microbial agents as an eco-friendly and effective approach. Numerous studies have been conducted in this field. Song et al. (2022) discovered that the application of organic matrix and composite microbial preparations in conjunction with straw return to fields offers substantial benefits in terms of organic matter accumulation and alteration of the soil microbial community. Deng et al. (2021) observed that combining microbial agents with plants markedly increased the content of organic matter, alkali-hydrolyzed nitrogen, and invertase activity in the soil. Sarangi et al. (2021) demonstrated that microorganisms could facilitate the decomposition of rice straw and bolster the stress resistance of rice. Furthermore, Zhou et al. (2014) reported that adding a microbial agent enhanced the release of P from pulverized rice straw. Collectively, these findings suggest that the use of microbial agents in straw return processes accelerates straw decomposition, improves soil conditions, and significantly boosts crop yields.

Microbial agents encompass a diverse group, including bacteria and fungi. Numerous studies have been conducted on *Bacillus subtilis*, a bacterial species, and *Trichoderma harzianum*, a fungal species, and their roles in agriculture. Applying *B. subtilis* enhances soil contents of available N, P, and K; promotes N, P, and K-fixing capacities (Chowdappa et al., 2013); and improves soil enzyme activity (Ng et al., 2022). In comparison to applications of 50% urea, employing a microbial agent formulated with *B. subtilis* has been shown to reduce soil nitrogen loss by 54% (Hermosa et al., 2012). Moreover, *B. subtilis* can promote crop growth while inhibiting the proliferation of pathogenic bacteria. This is due to the ability of *B. subtilis* to produce antimicrobial metabolites, which can be used as an alternative to synthetic chemicals or as a supplement to biopesticides for the control of plant diseases, in addition to the ability of *B. subtilis* to activate induced systemic resistance (ISR) in the plant, which increases plant resistance to pathogens, and ISR induces the synthesis of jasmonic acid and ethylene in the plant (Li et al., 2017). Chowdappa et al. (2013) demonstrated that *B. subtilis* inhibits the mycelial growth of tomato late blight, while significantly enhancing root crown growth, leaf area, and the seedling vitality index in tomatoes. In addition, the *B. subtilis* can secrete a variety of secondary metabolites that stimulate plant growth, increase disease resistance, and improve plant tolerance. A study found that cytokinins produced by *Bacillus megaterium* and *B. subtilis* on the surface of potato promote root cell growth and enhance root respiration, which in turn enhances the ability of the root system to absorb water, mineral elements, as well as increase potato tuber yield by increasing the net photosynthetic rate of potato leaves during the critical period of reproductive growth (Fan et al., 2021). Lima et al. (2019) revealed that inoculation of maize and soybeans with *B. subtilis* under water shortage conditions increases leaf water content and reduces leaf antioxidant activity to resist water stress. The effect of *T. harzianum* differs from that of *B. subtilis* in regulating the bacterial community and inhibiting pathogenic bacteria. It has been shown that seed germination and seedling growth rate of several crops, including wheat, tobacco, carrot and potato, were significantly enhanced and plant dry weight was increased by treatment with *T. harzianum* (da Silva Folli-Pereira et al., 2022), but too high a concentration may have an inhibitory effect (Gao et al., 2009). Abdenaceur et al. (2022) found that *T. harzianum* produces phosphatase and hydrogen cyanide, which contribute to promoting plant growth. Mironenka et al. (2021) reported that *T. harzianum* mitigates the adverse effects of the wheat pathogen *Fusarium* spp., and secretes cellulase to decompose organic matter. More than that, recent research advances have revealed that *T. harzianum* is able to enhance plant tolerance to abiotic stresses through interactions with plants (Yang et al., 2014; Lin et al., 2017). For example, treated cocoa seedlings not only grew faster but also demonstrated greater drought tolerance under drought conditions, which was reflected at the phenotypic, molecular and physiological levels of the plant. In addition, *T. harzianum* has the ability to be able to degrade straw. A research found that *T. harzianum* was able to produce cellulase, which was able to effectively degrade wheat straw (Radhakrishnan and Baek, 2017). While numerous studies have explored the functional mechanisms of *B. subtilis* and *T. harzianum* as individual microbial agents, research on the application of these two agents under straw-return conditions, especially in rice field soil environments, is still limited.

In this study, the effects of the combined application of *B. subtilis* and *T. harzianum* on the paddy soil environment, rice root system, straw decay, and rice yield were investigated to elucidate the underlying regulatory mechanisms of microorganisms in improving rice yield and soil fertility, facilitating efficient utilization of straw, and reducing environmental pollution. This study provides a theoretical and practical basis for green rice production.

## 2 Materials and methods

### 2.1 Experimental site and materials

The experiment was conducted in 2022-2023 at the Rice Research Institute of Sichuan Agricultural University in Wenjiang, Chengdu, Sichuan Province, China (30°43′N, 103°51′E), building upon previous experiments (Liu et al., 2019; Yao et al., 2020). The previous crop was wheat, which was naturally air-dried and rolled into small pieces (about 4 cm) at a moisture content of 15% for straw mulching. 4710 kg/hm^2^ of wheat straw (15.0% moisture content) was incorporated into the soil. The soil in the experimental field was sandy loam, with a topsoil depth of 0–20 cm, containing 2.26 g/kg of total N, 20.45 g/kg of organic matter, 115.82 mg/kg of available N, 28.19 mg/kg of available P, and 87.88 mg/kg of available K. The tested rice variety was “*Chuankangyou 2115*” (provided by Sichuan Agricultural University, a three-line hybrid rice variety with a total growth period of 151.8 d). The microbial agents used were *B. subtilis* and *T. harzianum* (both provided by Sichuan Green Microbial Technology Co., Ltd.) at a concentration of 200 × 10^9^ CFU/g.

### 2.2 Experimental design

The application rates of *B. subtilis* and *T. harzianum* were based on the method described by (Liu et al., 2018). The experiment used a completely randomized design with five treatments: T1 – no wheat straw return; T2 – return of wheat straw; T3 – return of wheat straw with a combined application of microbial agents (*B. subtilis*: *T. harzianum*=50 g: 50 g); T4 – return of wheat straw with a combined application of microbial agents (*B. subtilis*: *T. harzianum*=75 g: 25 g); and T5 – return of wheat straw with a combined application of microbial agents (*B. subtilis*: *T. harzianum*=25 g: 75 g). Three replicates were conducted in a plot with an area of 4.0 m × 5.0 m = 20.0 m². Wheat straw was fully incorporated into the soil, and one day before transplanting, a microbial agent was applied according to a prescribed ratio, with a total application rate of 50 kg/ha. On April 7, 2022, rice seeds were sown in a seeding field and covered with a film for seedling cultivation. By 15 May, the seedlings were transplanted to the experimental plots and planted at a spacing of 33.3 cm × 16.7 cm between rows and plants, one plant per hole. 12 rows with 29 plants per row were finally planted in each plot. Raised beds (40 cm wide) were constructed between plots and covered with a plastic film. Fertilization and irrigation were consistently managed, and measures were taken to prevent and control diseases, pests, and weeds. The total amount of N applied was 150 kg/ha, with compound fertilizer (Urea- Phosphorus pentoxide - Potassium oxide:15-15-15) applied at a ratio of 5:2:3 for basal, tillering, and panicle fertilization; basal fertilizer was applied 1 d before transplanting, tillering fertilizer was applied 7 d after transplanting, and panicle fertilizer was applied at the booting and heading stages in equal amounts.

### 2.3 Measurement items and methods

#### 2.3.1 Soil nutrients

At 15 d after transplanting, tillering stage (36 d after transplanting), jointing, heading, and maturing stages, five points were taken from each plot according to the diagonal line, and the soil total N, alkali-hydrolyzed N, available P, and available K were determined after drying and grinding through a sieve with a mesh aperture of 250 μm.

#### 2.3.2 Soil nutrients

Total N was determined using the Kaiser-type method, and available N was determined using the alkali-diffusion method; available P was determined using sulfuric acid-hydrochloric acid leaching spectrophotometry, and available K by ammonium acetate extraction using flame atomic absorption spectrophotometry (Bao, 2000).

#### 2.3.3 Straw decay rate

Using the nylon net bag method (Ocio et al., 1991), intact crop straw of similar thickness and length was selected and cut into 3–5 cm pieces. Exactly 30 g of the small pieces (N_0_) were weighed and placed in a net bag (25 cm x 35 cm, hole diameter of 425 μm nylon mesh bag), buried 5–10 cm deep in the tillage layer. At 15 d after rice transplanting, tillering stage, jointing stage, heading stage, and maturing stage, five samples were randomly selected from each plot, rinsed with tap water until the water was colorless, dried in an 80°C oven until they reached a constant weight, accurately weighed and the weight of each bag N_x_ was recorded. Straw decay rate W_x_ (%) = (N_0_ − N_x_)/N_0_ × 100.

#### 2.3.4 Rice root system

At the rice tillering, jointing, heading, and maturing stages, 30 rice plants were randomly selected from each plot to calculate the average tillering number of each plot, and then the sampling of plants was carried out according to the average tillering number of the plot; roots were sampled by taking a 33.3 cm × 16.7 cm × 30 cm soil core around the plant. Intact root systems were obtained by washing the roots under running water and placing them in nylon nets with a pore size of 0.4 mm. Root morphology parameters, such as total root length and root surface area, were measured using WinRHIZO Pro v.2009c software and an Epson Expression 10000XL scanner. Next, the roots were fixed at 105°C for 30 min and dried at 80°C to a constant weight. Single-stem root length (cm) = total root length per plant/total number of stems per plant; single-stem root surface area (cm2) = total root volume per plant/total number of stems per plant; the root dry weight per hectare (kg/hm2) = the product of the dry weight of a single-stem root system × the average number of tillers per plot × the basic seedling number.

#### 2.3.5 Soil enzyme activities

At 15 days after rice transplanting, tillering, jointing, heading and ripening stages, 5 points were selected from the diagonal of each plot and samples were collected at a depth of 0-20 cm using a soil sampler. After natural air drying, the soil samples were ground and screened with a diameter of 250 μm to determine the activities of sucrase, urease and acid phosphatase. Soil sucrase activity was determined using the 3,5-dinitro salicylic acid colorimetric method. Soil urease activity was determined using the phenol sodium hypochlorite colorimetric method, and acid phosphatase activity was determined using the sodium phenyl phosphate colorimetric method (Guan, 1986).

#### 2.3.6 Soil bacterial microbial community

2 g of fresh soil removed from rice roots, and immediately put into a sterile EP tube, frozen in liquid nitrogen, and stored at -80°C. DNA was extracted from soil samples using Soil DNA Kit (Omega), and then the bacterial 16S rRNA was amplified and sequenced by Shanghai Piceno Biotechnology Co., LTD. The sequences were compared in Silva library (https://www.arb-silva.de/) to determine the classification and abundance of strains. The Chao index represents the richness, and the higher the index value, the higher the richness of the community. Shannon index represents species diversity, and the higher the index value, the higher the diversity of the community. PCoA (Principal Coordinates Analysis), also known as principal coordinate analysis, is a non-binding data dimensionality reduction analysis method that can be used to study the similarity or divergence of sample community composition.

#### 2.3.7 Actual yield and its constituent factors

At the maturation stage, 50 plant-effective panicles were investigated in each plot, and 10 plants were randomly selected based on the average spike number. The evaluated traits included the number of empty grains, number of filled grains, and 1000-grain weight. Additionally, the seed-set rate and total floret number were calculated. The yield was calculated based on the actual number of plants at harvest (13.5 a moisture content).

### 2.4 Data analysis

Data were processed using Microsoft Excel 2019, and Sigma Plot 14.0 was used for graphical representation. Statistical analysis was performed using SPSS 24.0, one-way analysis of variance and Duncan’s multiple range test were used, and the least significant difference method was employed to determine significant differences between treatment means at P<0.05. Additionally, as the experimental results over two years show a consistent trend, the comprehensive report will primarily focus on the experiments conducted in 2022.

## 3 Results and analysis

### 3.1 Effects of microbial agents on rice yield and its components under rice-wheat rotation

**Table 1** shows that all treatments significantly affect rice yield and its components, except for grains per ear and 1000-grain weight in year 2022, and 1000-grain weight in year 2023. The results from the two-year experiment were consistent. Compared with T_1_ treatment, the rice yield of T_2_, T_3_, T_4_ and T_5_ treatment increased by 3.81%, 17.15%, 10.63% and 10.38% in 2022, and 12.75%, 26.63%, 15.45% and 13.76% in 2023, respectively. T3 treatment, which had the highest yield, was identified as the optimal treatment for combined microbial agent application under wheat straw return conditions. In 2022, from the perspective of yield components, the T_1_ treatment had a higher setting rate, which was significantly higher than that of other treatments, but this could not compensate for the lack of effective panicle number and total spikelet number. Compared to the T_1_ treatment, the T_2_ treatment significantly increased the number of spikelets per panicle by 4.26%; the application of microbial agents in treatments T_3_-T_5_ could further increase the effective panicle number and total spikelet number, showing a significant increase of 6.97 ~ 11.54% and 9.67 ~ 15.23% compared to T_1_, respectively. In 2023, compared to the T_1_ treatment, the T_2_ treatment significantly increased the effective panicle number by 9.85%; the combined application of microbial agents (T_3_-T_5_ treatments) significantly increased the effective panicle number by 3.86-33.12% and the total spikelet number by 3.87-23.74%, ensuring an increase in yield. Overall, the two-year data, compared with T_4_ and T_5_ treatments, T_3_ treatment could further increase the number of effective panicle and total spikelets of rice, thus increasing the yield of rice. In this study, T_3_ treatment combined with wheat stalk return to the field (*Bacillus subtilis: Trichoderma harzia* = 50 g: 50 g) was the appropriate treatment.

**Table 1:** Effects of combined application of microbial agents on rice yield and its components.

Season	Treatment	Effective panicle /(×10^4^/hm^2^)	Spikelets per panicle	Spikelets number /(×10^6^/hm^2^)	Seed-setting rate/%	1000-grain weight/g	grain yield /(kg/hm^2^)
**2022**	T_1_	227.73c	162.01a	368.95d	87.13a	35.33a	9270.37c
	T_2_	232.80c	165.60a	385.37c	84.97b	34.58a	9623.47c
	T_3_	254.00a	167.43a	425.15a	80.45c	34.44a	10860.02a
	T_4_	249.60ab	168.25a	419.77ab	81.29c	34.42a	10256.13b
	T_5_	243.60b	166.17a	404.62b	81.10c	35.08a	10232.76b
	***F* value**	20.87**	2.51	81.41**	20.55**	2.34	18.16**
**2023**	T_1_	186.60d	175.55b	327.74b	75.82b	33.88a	8482.05c
	T_2_	207.00b	168.76bc	349.36b	76.56b	34.57a	9436.26b
	T_3_	248.40a	163.35c	405.54a	81.59a	33.78a	10474.18a
	T_4_	208.80b	186.58a	389.62a	72.53c	34.00a	9638.25b
	T_5_	193.80c	175.67b	340.43b	76.57b	35.05a	9511.69b
	***F* value**	141.94**	10.51**	25.42**	12.73**	1.84	60.88**

Data in the same column with different letters are significantly different at the 5% level. *, ** indicate significant differences at the 0.05 and 0.01 levels, respectively. T_1_: no wheat straw return; T_2_: wheat straw return; T_3_: T_2_ + (Bacillus subtilis: Trichoderma harzianum=50:50); T_4_: T_2_ +(*Bacillus subtilis*: *Trichoderma harzianum*=75:25); T_5_: T_2_ + (*Bacillus subtilis*: *Trichoderma harzianum*=25:75).

### 3.2 Effects of microbial agent on rice root growth under rice-wheat rotation

As shown in **Tables 2** and **3**, the root morphology of rice followed a pattern of an initial increase before decreasing during the growth stages, peaking at the heading stage. During the tillering stage, the differences in single-stem root length and root surface area were not significant. However, in other growth stages, significant differences in these two indicators and population root dry weight were observed among the treatments. Compared to the T_1_ treatment, the T_2_, T_3_, T_4_, and T_5_ treatments increased the single-stem root length (RLS) by 0.88–1.99%, 4.77–10.38%, 5.18–7.77%, and 2.24–8.41%, respectively, from the jointing to the maturing stages. Single-stem root surface area (RAS) increased by 2.12–22.50%, 10.68–23.56%, 3.31–11.06%, and 3.18–15.87% in the T_2_, T_3_, T_4_, and T_5_ treatments, respectively, compared with the T_1_ treatment; the microbial agent-combined application treatments (T_3_–T_5_) showed the best performance regarding the RLS and RAS under the T_3_ treatment. During the tillering stage, the T_1_ treatment had the highest population root dry weight (RWP), significantly higher than the wheat straw return (T_2_) and microbial agent-combined application treatments (T_3_–T_5_). However, from the heading to maturing stages, the microbial agent-combined application treatments (T_3_–T_5_) were higher than the T_1_ treatment, with the T_3_ treatment showing the best performance, increasing RWP by 4.80–6.55% compared to the T_1_ treatment. In summary, while wheat straw return (T_2_) can promote root growth in rice from the jointing stage onwards, the combined application of microbial agents (T_3_–T_5_) based on straw return demonstrates a more pronounced effect, with the T_3_ treatment showing the most significant improvement in root growth.

**Table 2:** Effects of combined application of microbial agents on root morphology at the tillering and jointing stages.

Treatment	Tillering stage			Jointing stage		
	RLS/cm	RAS/cm^2^	RWP/(kg/hm^2^)	RLS/cm	RAS/cm^2^	RWP/(kg/hm^2^)
T_1_	403.13a	88.58a	431.00a	507.98b	103.98b	403.20b
T_2_	391.06a	86.52a	409.80b	518.10b	127.38a	438.40a
T_3_	402.47a	85.76a	366.40c	536.91a	128.47a	440.60a
T_4_	400.43a	84.96a	397.20b	536.71a	120.48a	410.80b
T_5_	399.62a	84.34a	366.40c	519.34b	108.93b	390.20c
**F value**	0.51	0.62	42.26**	12.31*	15.01**	46.83**

RLS, Single stem root length; RAS, Single stem root surface area; RWP, Population root dry weight. Data in the same column with different letters are significantly different at the 5% level. *, ** indicate significant differences at the 0.05 and 0.01 levels, respectively. T_1_: no wheat straw return; T_2_: wheat straw return; T_3_: T_2_ + (*Bacillus subtilis*: *Trichoderma harzianum*=50:50); T_4_: T_2_ +(*Bacillus subtilis*: *Trichoderma harzianum*=75:25); T_5_: T_2_ + (Bacillus subtilis: Trichoderma harzianum=25:75).

**Table 3:** Effects of combined application of microbial agents on root morphology at the heading and maturing stages.

Treatment	Heading stage			Maturing stage		
	RLS/cm	RAS/cm^2^	RWP/(kg/hm^2^)	RLS/cm	RAS/cm^2^	RWP/(kg/hm^2^)
T_1_	732.20b	151.56b	458.60d	688.14c	140.08b	427.40b
T_2_	773.02a	157.52ab	482.60b	694.19c	143.06b	456.00ab
T_3_	767.15a	167.75a	526.80a	759.58a	160.78a	477.20a
T_4_	770.11a	156.58ab	480.60bc	741.64b	155.57a	455.40ab
T_5_	756.20a	156.37ab	463.00cd	746.00b	158.98a	441.20b
**F value**	8.222*	1.64	21.54**	57.90**	15.96**	4.76*

RLS, Single stem root length; RAS, Single stem root surface area; RWP, Population root dry weight. Data in the same column with different letters are significantly different at the 5% level. *, ** indicate significant differences at the 0.05 and 0.01 levels, respectively. T_1_: no wheat straw return; T_2_: wheat straw return; T_3_: T_2_ + (*Bacillus subtilis*: *Trichoderma harzianum*=50:50); T_4_: T_2_ +(*Bacillus subtilis*: *Trichoderma harzianum*=75:25); T_5_: T_2_ + (*Bacillus subtilis*: *Trichoderma harzianum*=25:75).

### 3.3 Effects of microbial agent on straw decay and soil enzyme activities under rice-wheat rotation

**Figure 1:**
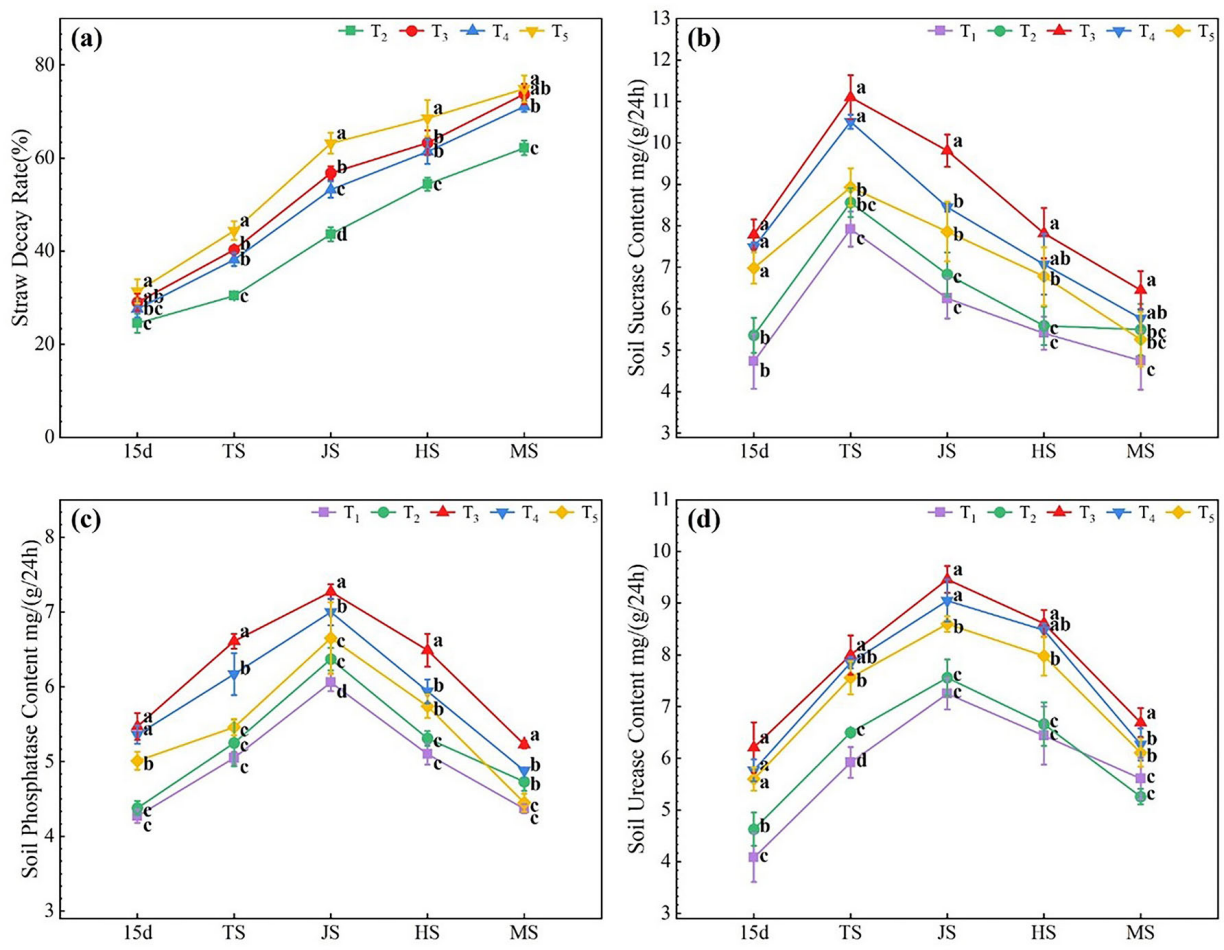
Effects of combined application of microbial agents on straw decay rate **(A)**, soil sucrase **(B)**, soil phosphatase **(C)** and soil urease **(D)** at main growth period of rice. 15d, at 15 days after rice transplanting; TS, tillering stage; JS, jointing stage; HS, heading stage; MS, Maturing stage. One-way analysis of variance and Duncan’s multiple range test were used. Different lowercase letters indicate significant difference in P<0.05 level between different treatments in the same growth period.

As shown in **Figure 1A**, the straw decay rate gradually increased with time, reaching 62.16–74.89% at the rice maturing stage. Compared to the T_2_ treatment, the microbial agent-combined application treatments (T_3_–T_5_) significantly increased the straw decay rate by 12.64–45.97% during all rice growth stages, with the T_5_ treatment showing the highest increase in straw decay rate, which was 1.54–10.12% and 5.10–15.75% more than that of the T_3_ and T_4_ treatments, respectively.

As shown in **Figure 1B**, soil sucrase activity in all treatments showed a similar trend, reaching a maximum at the tillering stage and then decreasing. Compared to the T_1_ treatment, the T_2_–T_5_ treatments increased soil sucrase activity by 3.33–13.39%, 35.79–64.62%, 21.40–58.21%, and 10.80–47.64%, respectively, with T_3_ treatment showing the most significant increase.

As shown in **Figure 1C**, the soil phosphatase activity in all treatments followed a trend of increasing and then decreasing, reaching a maximum during the jointing stage. Compared to the T_1_ treatment, the T_2_–T_5_ treatments increased soil phosphatase activity by 2.58–8.24%, 19.68–30.89%, 11.67–25.53%, and 1.83–17.33%, respectively, with the T_3_ treatment showing the most significant increase, followed by the T_4_ treatment; the T_5_ treatment showed no significant increase in the soil phosphatase activity.

As shown in **Figure 1D**, the soil urease activity of all treatments showed a similar trend of increasing and then decreasing, reaching a peak during the jointing stage. Compared to the T_1_ treatment, the T_2_ treatment significantly increased soil urease activity by 9.80–13.48% from 15 d after transplanting to the tillering stage, while the microbial agent-combined application treatments (T_3_–T_5_) significantly increased soil urease activity by 19.25–52.21%, 11.76–41.42%, and 8.91–37.25%, respectively, during all rice growth stages. These results indicate that wheat straw return combined with microbial agent application can increase the straw decay rate and soil enzyme activity; the T_5_ treatment showed the best effect on straw decay, the T_3_ treatment showed the best effect on soil sucrase and phosphatase activities, and the T_3_–T_5_ treatments showed a significant effect on soil urease activity, with T_3_ > T_4_ > T_5_.

### 3.4 Effects of microbial agents on soil available nutrients under rice-wheat rotation

**Figure 2:**
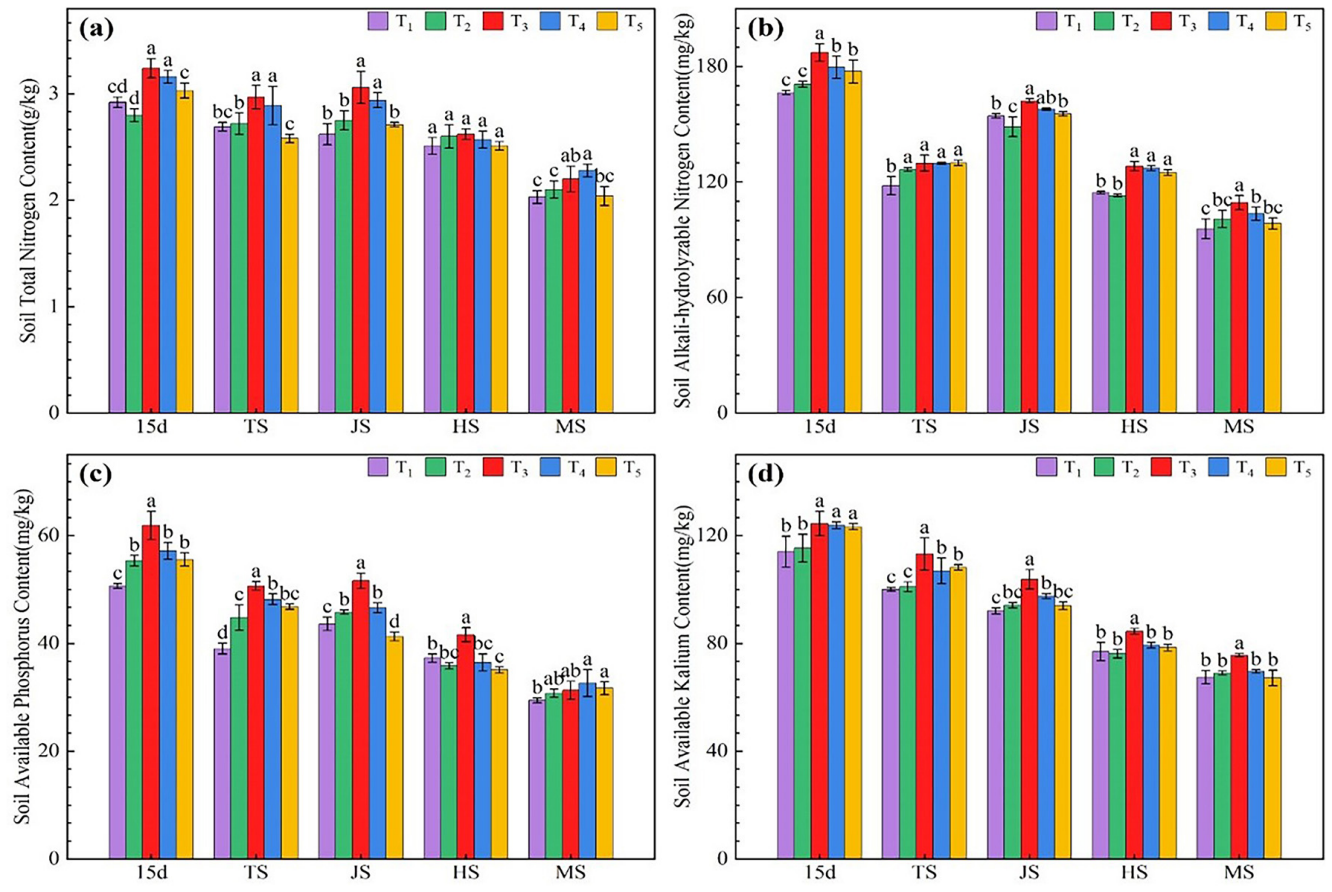
Effects of combined application of microbial agents on soil total nitrogen content **(A)**, soil alkali-hydrolyzed nitrogen content **(B)**, soil available phosphorus content **(C)** and soil available kalium content **(D)** at main growth period of rice. 15d, at 15 days after rice transplanting; TS, tillering stage; JS, jointing stage; HS, heading stage; MS, Maturing stage. One-way analysis of variance and Duncan’s multiple range test were used. Different lowercase letters indicate significant difference in P<0.05 level between different treatments in the same growth period.

As shown in **Figure 2A**, except for the heading stage, the total soil N of the T_3_ and T_4_ treatments was significantly higher than that of the T_1_ treatment during all other growth stages, with increases ranging from 8.54% to 16.92% for T_3_ and from 7.31% to 12.48% for T_4_. There was no significant difference in total soil N between the T_2_ and T_5_ treatments and the T_1_ treatment during all rice growth stages. According to **Figure 2B**, the T_2_ treatment of straw returning was significantly higher than T_1_ in TS; the microbial agent-combined application treatments (T_3_–T_5_) significantly increased soil available nitrogen content compared with T_1_ and T_2_ at day 15 and HS (**Figure 2B**). T_3_ treatment showed the highest content in all periods, which significantly increased by 4.85%-12.46% compared with T_1_. These results indicate that combining microbial agents can significantly increase the soil available N content, with T_3_ showing the best effect, followed by T_4_. The soil available N content showed a decreasing trend during the rice growth stages, and the decrease in the soil available N content of the combined microbial agent application treatments (T_3_–T_5_) was higher than that of the T_1_ treatment, with the T_3_ treatment showing the most significant decrease. As shown in **Figure 2C**, the available phosphorus content in soil treated with microbial agents (T_3_-T_5_) was significantly higher than that in the T_1_ treatment from 15 d after transplanting to TS, with the T_3_ treatment showing the greatest increase, which was 6.35 ~ 29.81% higher than the T_1_ treatment. Compared to the T_1_ treatment without straw return, the soil available phosphorus content in the straw return T_2_ treatment significantly increased during the periods 15 days after transplanting, TS, and JS. Within the treatments using microbial agents (T_3_-T_5_), the T_3_ treatment further increased the soil available phosphorus content compared to T_2_. The T_5_ treatment, which involved the application of microbial agents, showed the lowest soil available phosphorus content during the JS and HS periods. Soil available K (**Figure 2D**) in all treatments showed a decreasing trend during all rice growth stages. Except during the HS period when the available potassium content in the soil of T_1_ was higher than that of T_2_, the available potassium content in T_2_ was higher than that in T_1_ during the rest of the periods. However, it is important to note that the differences in soil available potassium content between T_1_ and T_2_ were not significant in any of the growth stages. The available potassium content in the soil of the microbial agent combined treatment (T_3_-T_5_) was significantly higher than that in treatments T_1_ and T_2_ at 15 days after transplanting and during the TS period; the T_3_ treatment showed the highest available potassium content in the soil at all growth stages, which was 9.15 ~ 13.14% higher than that in treatments T_1_ and T_2_.

### 3.5 Effects of microbial agent on soil bacterial diversity in rice heading stage under rice-wheat rotation

**Figure 3:**
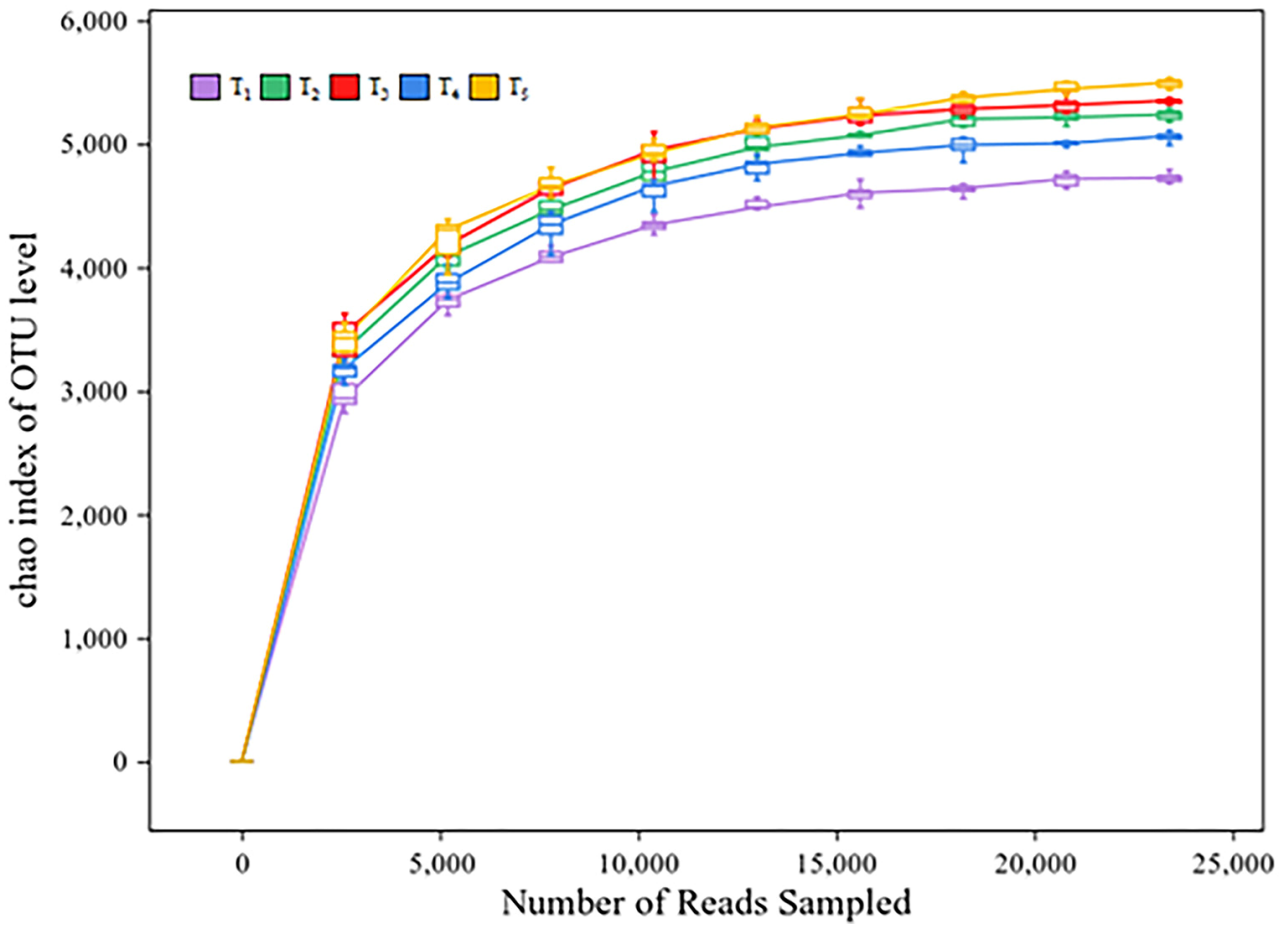
Dilution curves for different treatments. The abscissa represents the amount of sequencing data; The ordinate indicates the number of species.

**Figure 4:**
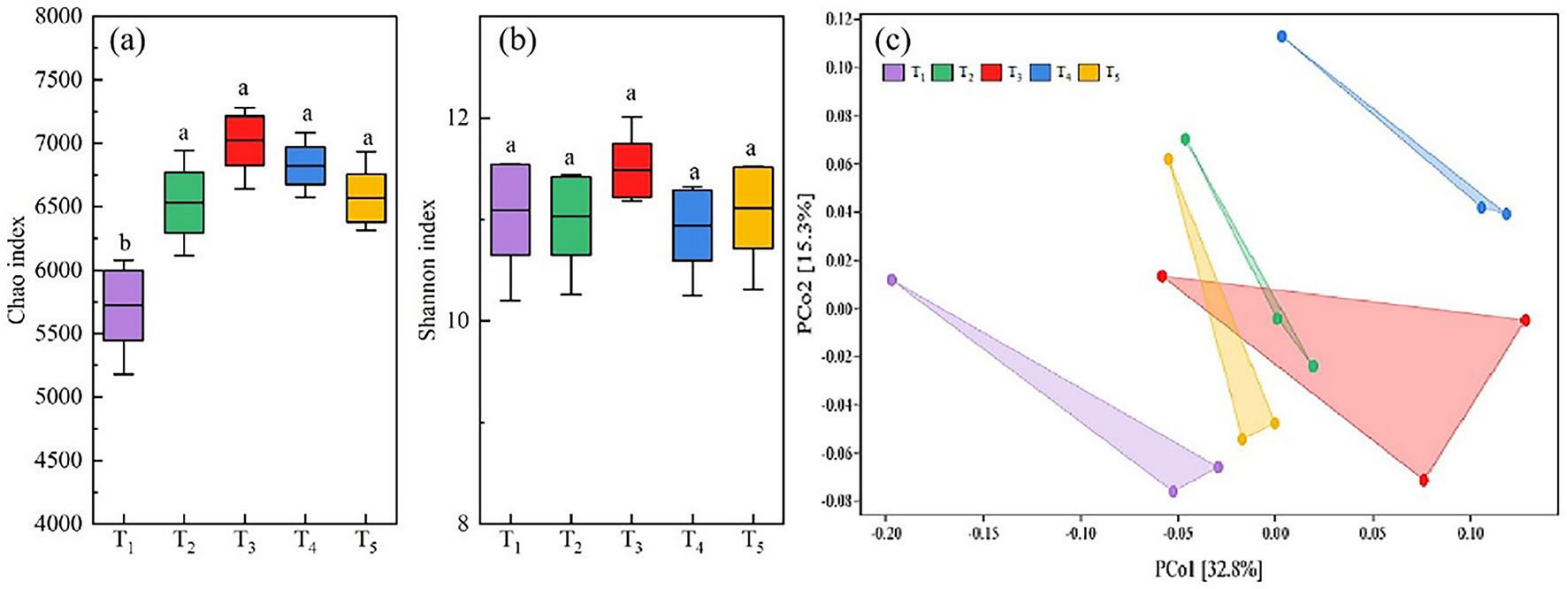
Changes in chao index **(A)** for alpha diversity and shannon index **(B)** for beta diversity of soil bacteria at the OTU level. Beta diversity using Primary coordinate Analysis (PCoA) based on OUT Level weighted UniFrac distance measures **(C)**. One-way analysis of variance and Duncan’s multiple range test were used. Different lowercase letters indicate significant difference between different treatments at P<0.05 level.

A total of 3,363,677 valid reads and 93,761 OTUs were obtained by 16S rRNA amplicon sequencing of soil bacteria. The average amplicon length was 420 bp. The number of valid sequences detected in each soil sample exceeded 40,000, and the sparse curve was flat, indicating that the genetic data was sufficient to reasonably estimate bacterial OTUs in the soil samples (**Figure 3**). The analysis of microbial community diversity index of each treatment found that the Alpha diversity Chao richness index (**Figure 4A**) showed significant differences between T_1_ and T_2_, T_3_, T_4_ and T_5_ among treatments, while the Shannon diversity index (**Figure 4B**) showed no significant differences among treatments. PCoA was used to analyze the relative contributions of straw returning to field and microorganisms to soil bacterial diversity. From the **Figure 4C**, it can be observed that there is a certain degree of separation among the soil bacterial communities across different treatments. The community in T_1_ (no straw return to the field) is significantly separated from the other treatments along the PCo1 axis, indicating that the soil bacterial community composition without straw return differs greatly from those with straw return. Additionally, T_2_ (straw return) compared to treatments with different proportions of microbial inoculants (T_3_-T_5_) shows that the latter are closer to each other but also display some degree of separation, especially in the T_4_ treatment, which was significantly different from T_3_ and T_5_ in that it was biased more upwards in the PCo2. Thus, this suggests that the application of microbial agents alters the soil bacterial community and that different microbial agent ratios do alter the bacterial community of the soil. The proximity or overlap of T_3_, T_4_, and T_5_, which involve different proportions of microbial inoculants, in the PCoA plot may indicate that although the proportions of the inoculants differ, the trend in their impact on the bacterial community might have certain similarities. Interestingly, the distance between T_3_ and T_4_ treatments and T_2_ (straw return) is closer, whereas T_5_ is further from T_2_, indicating that T_5_ may have a more distinct microbial community change.

### 3.6 Effects of microbial agent on composition of soil bacterial community in rice heading stage under rice-wheat rotation

**Figure 5:**
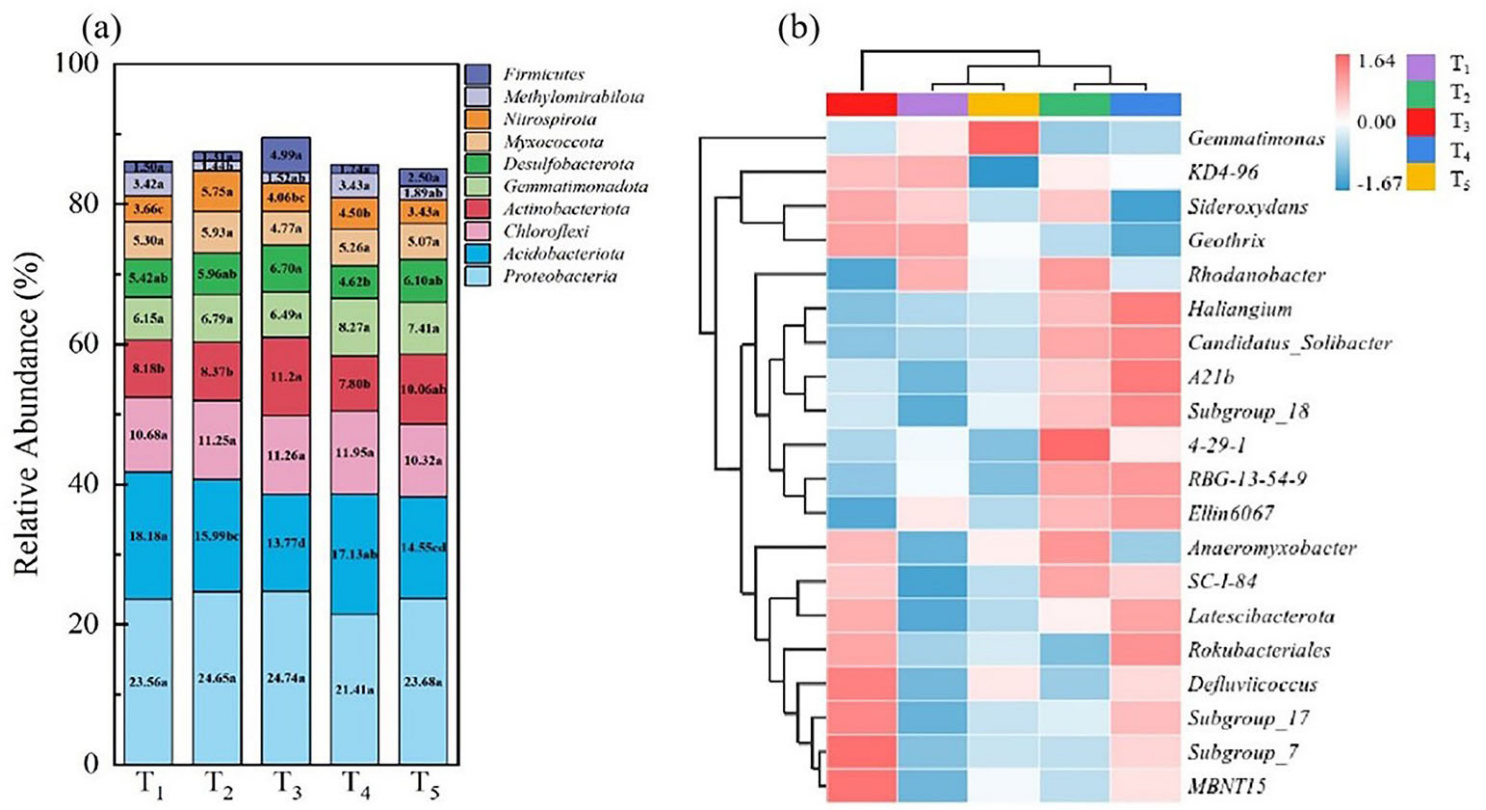
Heatmap analysis of the relative abundance of bacteria at the level of phylum **(A)** and species **(B)** under different treatments. Different colours in **(A)** refer to different bacterial phylums. The red colour in **(B)** refers to the increase in relative abundance and the blue colour indicates the decrease in relative abundance; the shade of the colour represents the degree of increase or decrease.

**Figure 5A** shows that the top 10 bacterial phyla include *Proteobacteria*, *Acidobacteriota*, *Chloroflexi*, *Actinobacteriota*, *Gemmatimonadota*, *Chloroflexi Desulfobacterota*, *Myxococcota*, *Nitrospirota*, *Methylomirabilota*, and *Firmicutes*. In **Figure 5A**, the average proportion of total soil bacteria among different treatments was 85.56%. Some bacterial groups such as *Proteobacteria*, *Chloroflexi*, and *Myxococcota* maintained stable abundance at the phylum level, but the relative abundance of *Actinobacteriota* in treatment T_3_ was higher than the other treatments, and reached significant levels with treatments T_1_, T_2_ and T_4_.

To further compare the differences in genera composition between samples and illustrate the trends in species abundance distribution, a heatmap analysis was conducted. A clustered heatmap was drawn using the abundance data of the top 20 genera in terms of average abundance (**Figure 5B**). Soil bacteria under different treatments exhibited different abundances at the genus level. Compared to T_1_, treatment T_2_ had higher abundances of *4-29-1* and *Anaeromyxobacter*; in treatments T_3_-T_5_, where microbial agents were applied, the abundances of *Defluviicoccus* and *MBNT15* were relatively higher than in T_1_ and T_2_; compared to T_1_ and T_2_, treatment T_3_ had a higher abundance of *MBNT15*, *Defluviicoccus* and *Rokubacteriales.*

### 3.7 Correlation analysis between root traits and yield and its components

**Figure 6:**
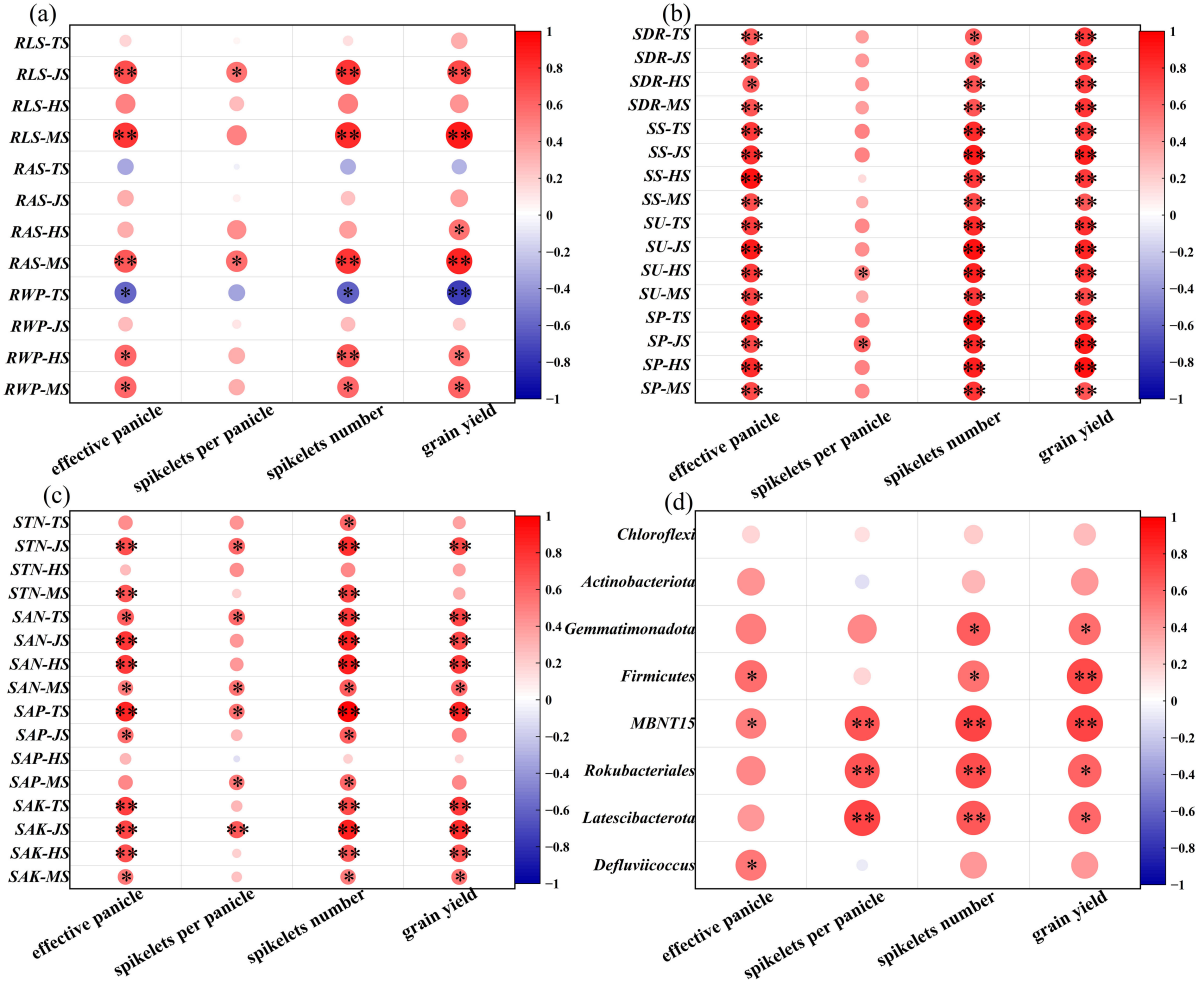
Relationship between root morphology **(A)**, straw decay and soil enzyme activity **(B)**, soil nutrient characteristics **(C)**, microbial at phylum level **(D)** and yield characteristics under combined application of microbial agent. TS, Tillering stage; JS, Jointing stage; HS, Heading stage; MS, Maturing stage; RLS, RLS: Single stem root length; RAS, Single stem root surface area; RWP, Population root dry weight; SDR, Straw decay rate; SS, Soil sucrase; SU, Soil urease; SP, Soil phosphatase; STN, Soil total nitrogen; SAN, Soil alkali-hydrolyzable nitrogen; SAP, Soil available phosphorus; SAK, Soil available kalium; *, ** indicate significant differences at the 0.05 and 0.01 levels. Red means increased abundance, blue means decreased abundance; The darker the color, the greater the increase/decrease.

There was no significant correlation between RLS and RAS and yield components at TS, while there was significant negative correlation between RWP and effective panicle, spikelets number and total yield (**Figure 6A**). However, RLS, RAS and RWP were positively correlated with effective panicle, spikelet number and yield at maturity stages. In addition, RLS was positively correlated with effective panicle number, spikelets number and yield during the whole growth period. In conclusion, root quality and morphological traits significantly affected the increase of effective panicle number and effective spikelets after wheat straw was returned to the field, which ensured the increase of yield.

SDR is significantly positively correlated with effective panicle, spikelets number, and yield during all stages (**Figure 6B**). **Figure 6B** also shows that during the tillering and jointing stages, SS, SU, and SP are significantly positively correlated with the effective panicles and spikelets number and yield. As shown in **Figure 6C**, during the jointing and maturity stages, STN is highly significantly positively correlated with the effective panicles and spikelets number; SAN and SAK are significantly positively correlated with the effective panicles, the spikelets number, and yield at all stages; SAP is mainly significantly positively correlated with the effective panicles, spikelets per panicle, spikelets number, and yield during the heading stage. In **Figure 6D**, during the heading stage, soil microorganisms at the phylum level are mostly positively correlated with yield, with *MBNT15*, *Rokubacteriales*, and *Latescibacterota* genera showing significant positive correlations with the spikelets per panicle, spikelets number, and yield.

## 4 Discussion

### 4.1 Effects of microbial agent on wheat straw decay and soil enzyme activity under wheat straw return

In our study, the combined application of microbial agents under straw returning can significantly enhance the root growth, straw decomposition, soil available nutrients from jointing to heading stage of rice, and improve soil microbial community at heading stage of rice, contributing to a significant increase in the number of effective panicles and total spikelets, thereby increasing rice yield.

Crop straw is a valuable and recyclable biological resource, rich in cellulose, lignin, N, P, K, and trace elements (Mishra et al., 2023). The decay of crop straw is a complex biochemical process in which microorganisms play important roles. Extensive studies have been conducted on the microbial decay of straw. Liu et al. (2016) showed that microbial agents accelerated the decay rate of wheat straw in the soil and influenced its final mineralization degree. Mehmood et al. (2020) and Moon (2022) also proved that adjusting the microbial diversity in soil by increasing certain microbial members is an effective method to improve the efficacy of soil-beneficial flora, which can improve the decomposition of substances such as straw, promote the removal of harmful substances and improve soil fertility. These studies indicated that microbial agents can significantly promote the decay of crop straw and nutrient release. This is consistent with the results of this study, where straw return to the field (with microbial inoculant application) treatments T_3_-T_5_ showed significantly enhanced straw decomposition and nutrient release compared to the straw return treatment without inoculant (T_2_). However, it is important to note that different ratios of microbial inoculants had varying effects on straw decomposition (Li et al., 2024). Our study indicates that the decomposition rate of wheat straw was highest under the T_5_ treatment, with a microbial agent application ratio of *B. subtilis* (25 g): *T. harzianum* (75 g). This suggests that a 1:3 ratio might be more suitable for straw decomposition. This may be due to the fact that *T. harzianum* secretes cellulase, which plays a more critical role in straw decomposition (Li et al., 2016).

This is consistent with the results of this study, where straw return to the field (with microbial agent application) treatments T_3_-T_5_ showed significantly enhanced straw decomposition and nutrient release compared to the straw return treatment without microbial agent (T_2_). However, it is important to note that different ratios of microbial agent had varying effects on straw decomposition. Our study indicates that the decomposition rate of wheat straw was highest under the T_5_ treatment, with a microbial agent application ratio of 25 g:75 g. This suggests that a 25:75 ratio might be more suitable for straw decomposition. This may be due to the fact that a significant amount of *T. harzianum* secretes more cellulase, playing a more crucial role in straw decomposition (Li et al., 2016).

Soil enzymes act as catalysts for biochemical reactions and are closely related to the physicochemical properties, nutrient status, and biological activity of the soil. These enzymes are essential indicators for evaluating soil fertility and environmental quality (Marx et al., 2001; Chen et al., 2018; Humberto et al., 2020; Hu et al., 2022). Zhang et al. (2023) showed that the application of exogenous microbial agents reshaped the rhizosphere bacterial community structure of bananas and improved the activity of soil enzymes, such as soil catalase and dehydrogenase. Notably, different agents have varying effects.

Ma et al. (2023) applied four kinds of bacteria (*XF-5C*, *Clostridium L13*, *Bacillus licheniformis PB3*, *Methylbacterium B0021*), the four microbial agents significantly increased the activities of soil urease, soil phosphatase and soil peroxidase to varying degrees, *XF-5C* and *L13* significantly increased soil urease activity, while *B0021* significantly increased soil phosphatase activity. Liu A. et al. (2022) demonstrated that after the application of *Bacillus*, the activities of sucrase, urease, phosphatase, and other enzymes in the soil were significantly improved. Gong et al. (2013) also found that inoculating *T. harzianum* in rice seedbeds can enhance the activity of soil urease, sucrase, and phosphatase. The results of our study are consistent with previous findings, showing that the soil enzyme activity in the straw return treatment T_2_ was relatively higher compared to the no straw return treatment T_1_. Moreover, upon the application of different ratios of *B. subtilis* and *T. harzianum* on top of straw return, the activities of soil sucrase, phosphatase, and urease in all treatment groups were further increased to varying degrees. However, the degree of improvement varied with different ratios, with the best being a *B. subtilis* to *T. harzianum* ratio of 1:1 (T_3_). The application of microbial agents can increase the activity of soil enzymes, and the increased activity of soil enzymes can provide more nutrients for the growth of microorganisms, thus promoting the reproduction of microorganisms, which can form a kind of benign cycle for soil ecology (Wu et al., 2023a). Therefore, microbial agents can be used to regulate soil microbial diversity. In this experiment, all three ratios of microbial agents improved soil enzyme activity in the rice field, with a *B. subtilis*: *T. harzianum* of 50 g: 50 g being the most effective in enhancing soil enzyme activity. This experiment was conducted in the ecological area of Sichuan, with a wheat straw return rate of 4710 kg/hm^2^ and a water content of 15.0% for agent application. However, the suitability of the optimal microbial agent ratio identified in this study for different ecological areas and soil conditions remains to be further validated.

### 4.2 Effects of microbial agent application on rice root system, soil nutrients, and yield under wheat straw return

This study showed that applying microbial agents could accelerate straw decay, increase soil nutrients, and promote the growth of rice root systems, thereby improving rice yields. It is noteworthy that the root phenotypes of the wheat straw-returned treatment (T_2_) were mostly weaker than those of the non-returned treatment (T_1_) during the early stages of rice growth. The reason for this phenomenon may be due to the incomplete decomposition of wheat straw in the early stage affecting the root growth, while the root phenotypes were lower in the straw-returned treatment with the application of microbial agents (T_3_-T_5_) compared to the wheat straw-returned treatment (T_2_), which may be due to the fact that nutrients in the wheat straw stimulate the activity of soil microorganisms (Guo et al., 2015), increasing the nutrient conversion rate of soil microorganisms, which results in microorganisms competing for nutrients with the rice root system to compete for nutrients, temporarily inhibiting the growth of the rice root system (Singh et al., 2004).

In addition, root length, root surface area and root dry weight were higher in the wheat straw-returned treatment (T_2_) than in the control (T_1_) during the remaining growth period due to nutrient release from the decomposition of wheat straw; and some of the root phenotypes were further increased in the microbial agents applied on top of the wheat straw-returned treatment (T_3_-T_5_).This is consistent with the results of Xu et al. (2018), indicating that adding microbial agents could promote plant growth, possibly because the beneficial flora in the microbial inoculants improved the microbial community in the soil (Liu L. et al., 2022). The application of microbial agents increased soil enzyme activity, making nutrients in the soil more easily absorbed by rice and promoting nutrient release from straw decay (Wu et al., 2023b), which is beneficial for the growth of the rice root system. The results of this experiment showed that rice yield, effective panicles, spikelet number, single-stem root length, single-stem root surface area, and root dry weight were significantly correlated. This is consistent with the results of Singh et al. (2004), indicating that better root morphology can contribute to the formation of effective panicles and spikelet numbers, thereby benefiting yield.

This study demonstrated that the application of microbial agents in combination wheat straw return could increase the soil nutrient content, especially before the jointing stage of rice growth. Chen et al. (2021) reported that microbial agents can increase the content of available N and P in soil; Wang et al. (2023) found that microbial agents can not only promote the proliferation of beneficial microorganisms in continuous cropping soil but also change the physical and chemical properties of the soil, such as increasing available phosphorus. Furthermore, Wu and Lin (2003) revealed that microorganisms have a unique impact on nutrient transformation and can release nutrients from insoluble mineral substances, as observed with microbial agents like *Bacillus* spp. and *Pseudomonas* spp. with phosphate-solubilizing capabilities. This indicates that microbial agents can convert soil-insoluble nutrients into available nutrients that are easily absorbed by plants, thus promoting plant growth, and this finding is similar to the results of this study. In addition, the soil nutrient results of this experiment showed that wheat straw return could significantly increase the available N and P in the soil, which differs from the results of Liu et al. (2018) and may be attributable to the type of straw and the soil environment. Wang (2006) reported that the combination of microbial residue and organic fertilizer resulted in higher effective panicles and grain-setting rates, showing an increase of 9.30% in effective panicles and 16.66% in grain-setting rate compared to conventional fertilization, indicating that microbial residue combined with organic fertilizer can stabilize tillering and promote panicles; which is consistent with the results of this study, where the application of microbial agents increased the number of effective panicles by 6.97–11.54%. The results of this experiment indicated that the T3 treatment (50g of *B. subtilis* and *T. harzianum* respectively) yielded the best results, significantly increasing soil nutrient content, improving root dry weight, optimizing root morphology, promoting effective tillering of rice, and consequently increasing yield.

### 4.3 Effects of microbial agent application on soil bacteria at heading stage of rice under wheat straw return

Rhizosphere soil microbial community is one of the indexes to evaluate soil quality. In general, the richer the soil microbial species and the more active the metabolism, the healthier the soil is and the more suitable it is for plant growth (Xue et al., 2011). The role and application of microorganisms in agricultural production have been increasingly recognized. The booting stage of rice is a critical period for its nutritional growth and coincides with the peak demand for water and nutrients. Analyzing bacterial communities in the soil during this stage is essential for understanding the structure of microbial communities in the rhizosphere of rice (Wei et al., 2010). In this study, there were no significant differences in the Simpson index among different treatments, but the Chao1 index was lowest in the T_1_ treatment, indicating a significant difference from other treatments. This suggests that straw return and microbial inoculant application can affect the diversity and richness indices of bacterial communities in the rhizosphere of rice, impacting the microbial community structure. The results of the phylum-level richness index shown that the dominant phyla are *Proteobacteria*, followed by *Acidobacteria*, *Chloroflexi*, *Actinobacteria*, and *Firmicutes*. Previous research has indicated that *Proteobacteria* generally thrive in nutrient-rich environments, suggesting that the application of microbial agents has increased the nutrient content in the soil (Solank et al., 2020). Moreover, *Acidobacteria*, an important group of bacteria in soil, involved in the degradation of plant residues, iron cycling, one-carbon compound metabolism, and photosynthesis, contributing to material cycling and ecological environment construction (Wang et al., 2016). Research by Li et al. (2020) on the combined application of organic fertilizers also showed that the most abundant phyla under different treatments are *Proteobacteria*, *Chloroflexi*, and *Actinobacteria*. Additionally, variations in geographic location, soil types, and cultivation methods can lead to differences in bacterial communities in rice fields. Among several treatments, T_3_ had the highest yield and, compared to other treatments, also had the highest abundance of *Actinobacteria*, which are widely considered biocontrol agents for plants and effective against rice sheath blight (Wang et al., 2012). Heatmap analysis indicates that fertilization methods can influence the abundance of microbial communities in the rhizosphere soil, with differences observed at the genus level among treatments. The genus *MBNT15* was dominant in the T_3_ treatment, and its role in enhancing rice production requires further research.

## 5 Conclusion

From the results of this experiment, it can be concluded that returning wheat straw to the soil can effectively increase both the alkali-hydrolyzed N and available P in the soil, compared to no returning wheat straw. The application of microbial agents to the returned wheat straw can accelerate straw decay, increase soil enzyme activity and nutrient content, promote the growth of the rice root system, and ultimately increase rice yield. Furthermore, the combination of 50 g of *B. subtilis* and 50 g of *T. harzianum*, applied with a full return of wheat straw, showed the best effect. The dominant bacteria genera of T_3_ treatment were *MBNT15*, *Defluviicoccus*, *Rokubacteriales* and *Latescibacterota*. Correlation analysis revealed that soil microorganisms present during the heading stage were significantly positive correlated with yield at the phylum level, including *Gemmatimonadota* and *Firmicutes*. There was a positive correlation between the genus level and the yield of *MBNT15*, *Rokubacteriales* and *Latescibacterota*. In addition, correlation analysis indicated that the straw decay rate, the group root dry weight, soil enzyme, and nutrient content were all positively correlated with effective panicles and total spikelet number of rice, particularly the high group root dry weight of rice after jointing. A high wheat straw decay rate, soil enzyme activity, and enhanced nutrient content from tillering to jointing are key factors in increasing effective panicles and total spikelet number, consequently leading to an increase in rice yield.

## Data availability statement

The original contributions presented in the study are included in the article/supplementary material. Further inquiries can be directed to the corresponding author.

## Author contributions

YW: Writing – original draft, Methodology, Investigation. YM: Software, Formal analysis, Writing – original draft, Methodology, Investigation. ZW: Writing – review & editing, Software, Data curation. YY: Writing – review & editing, Software, Data curation. XY: Writing – review & editing, Visualization. KC: Writing – review & editing, Visualization. YL: Writing – review & editing, Visualization. ZH: Writing – review & editing, Formal analysis. XH: Writing – review & editing, Formal analysis. PD: Writing – review & editing, Formal analysis. CL: Writing – review & editing, Software. ZY: Writing – review & editing, Supervision. ZC: Writing – review & editing, Supervision. JM: Writing – review & editing, Supervision. YS: Writing – review & editing, Writing – original draft, Visualization, Supervision, Project administration, Methodology, Investigation, Funding acquisition, Formal analysis, Data curation, Conceptualization.
